# Diagnosis and treatment of polycythemia vera: Brazilian experience from a single institution

**DOI:** 10.1590/S1516-31802008000100010

**Published:** 2008-01-03

**Authors:** Camila da Cruz Gouveia Linardi, Luís Fernando Pracchia, Valeria Buccheri

**Keywords:** Polycythemia vera, Treatment outcome, Thrombosis, Survival, Brazil, Policitemia vera, Tratamento, Trombose, Sobrevivência, Brasil

## Abstract

**CONTEXT AND OBJECTIVE:**

Polycythemia vera (PV) is a chronic myeloproliferative disorder characterized by predominant proliferation of erythroid precursors. Few data are available concerning Brazilian patients with this condition. The aim of this study was to describe clinical and demographic characteristics of PV patients at diagnosis and analyze their long-term outcomes.

**DESIGN AND SETTING:**

Retrospective study at the Division of Hematology, Faculdade de Medicina da Universidade de São Paulo (FMUSP), São Paulo.

**METHODS:**

All consecutive patients with PV diagnosed according to World Health Organization criteria were eligible for this study. Clinical and demographic characteristics, thrombotic events, transformation to acute leukemia, myelofibrosis and survival were evaluated.

**RESULTS:**

Sixty-six patients were evaluated. Thirty-six (54.5%) were females, with a median age at diagnosis of 61 years. At diagnosis, the median hemoglobin concentration was 18.8 mg/dl and the median platelet count was 593,000/mm^3^. Fifty-eight patients (88.0%) were treated with hydroxyurea with or without phlebotomy. During a median follow-up of 77 months, 22 patients (33.3%) had new thrombotic events, mainly of arterial type. The overall incidence of leukemia and myelofibrosis was 0.42% per patient-year and 1.06% per patient-year, respectively. Median overall survival was not reached and the seven-year survival rate was 77.8%.

**CONCLUSION:**

The PV patients described here had long survival and arterial thrombotic events were the most important and common complication among this population.

## RESUMO

**CONTEXTO E OBJETIVO:**

A policitemia vera (PV) é uma doença mieloproliferativa crônica, caracterizada pela proliferação de precursores hematopoéticos, principalmente da série eritróide. Poucos dados são disponíveis sobre pacientes brasileiros portadores desta doença. O objetivo do presente estudo é analisar as características de pacientes portadores de PV ao diagnóstico e a sua evolução clínica a longo prazo.

**TIPO DE ESTUDO E LOCAL:**

Estudo retrospectivo unicêntrico, realizado no Serviço de Hematologia da Faculdade de Medicina da Universidade de São Paulo.

**MÉTODOS:**

Foram elegíveis para este estudo os pacientes com PV diagnosticados de acordo com os critérios estabelecidos pela Organização Mundial da Saúde. Foram avaliadas as características demográficas e clínicas ao diagnóstico, as complicações trombóticas, a transformação para leucemia aguda e mielofibrose e a sobrevida.

**RESULTADOS:**

Foram avaliados 66 pacientes; 36 (54,5%) eram do sexo feminino, com uma mediana de idade ao diagnóstico de 61 anos. As medianas da concentração de hemoglobina e da plaquetometria ao diagnóstico foram de 18,8 mg/dl e 593.000/mm^3^, respectivamente. 58 (88,0%) foram tratados com hidroxiuréia associada ou não à flebotomia. Em uma mediana de acompanhamento de 77 meses, 22 (33,3%) pacientes apresentaram eventos trombóticos, predominantemente arteriais. A incidência de leucemia e mielofibrose foi de 0,42/100 pacientes-ano e 1,06/100 pacientes-ano, respectivamente. A mediana de sobrevida global não foi atingida, a taxa de sobrevida em sete anos foi de 77,8%.

**CONCLUSÃO:**

Os portadores de PV em nosso serviço apresentaram longa sobrevida. Os eventos trombóticos arteriais foram a principal complicação da população estudada.

## INTRODUCTION

Polycythemia vera (PV) is a clonal chronic myeloproliferative disease that affects mainly elderly individuals and is characterized by cytokine-independent proliferation of myeloid precursors. This mainly affects the erythroid lineage and leads to excessive accumulation of erythrocytes in peripheral blood. Many patients also present increased circulating granulocytes and platelets. The prevalence of PV in Brazil is not accurately known and there are no published data concerning outcomes among PV patients in our country.

The diagnosis of PV is established by means of strict criteria that allow secondary causes of polycythemia to be ruled out. Formerly, the diagnosis was made by means of the diagnostic criteria established in the late 1960s by the Polycythemia Vera Study Group (PVSG).^1^ In 2001, the World Health Organization (WHO)^2^ proposed a new set of diagnostic criteria for PV that incorporate the acquired knowledge on the pathological and genetic features of the disease.

The long-term outcomes among PV patients are quite variable. Because of erythrocytosis and thrombocytosis with associated microvascular disturbances and increased blood viscosity, PV patients generally have a predisposition towards arterial and venous thrombosis. These thrombotic complications are the main causes of death and morbidity.^3^ There is also a risk of progression to secondary myelofibrosis (sMF) or secondary acute myeloid leukemia (sAML), with little data regarding the predisposing factors involved in the pathogenesis of PV.^4^

There are no curative therapies for PV and its treatment has the objective of reducing symptoms and the risk of thrombosis, and avoiding the hematological transformation to sAML. Patients whose peripheral erythrocyte counts are under adequate control have low mortality rates, with 80% of them surviving for more than 12 years.^3,4^

The current available therapies are phlebotomy and myelosuppressive drugs. The use of alkylating agents such as chlorambucil, busulfan and pipobroman, or therapy with radioactive ^32^phosphorus can increase the risk of sAML in comparison with phlebotomy alone.^5^ The use of the non-alkylating agent hydroxyurea, with or without phlebotomy, can promote good hematological control, and today this is the drug most used for managing PV patients.^6^ Increased risk of leukemic transformation was not observed with the use of hydroxyurea in retrospective studies.^4,7,8^ Additionally, patients who are exclusively treated with phlebotomy have more cardiovascular events than do those treated with myelosuppressive drugs supplemented with phlebotomy.^5^

Another drug used for treating PV, particularly among young or pregnant patients, is alpha-interferon (INF). Its use may be accompanied by side effects that are not easily tolerated by many elderly patients.^9^

## OBJECTIVE

The aim of this study was to describe the clinical and demographic characteristics of PV patients at diagnosis and analyze their long-term outcomes.

## MATERIALS AND METHODS

### Patients

All consecutive patients with chronic myeloproliferative disease that fulfilled the WHO diagnostic criteria for PV were eligible for this retrospective study. All these patients were treated in the Hematology Division of Hospital das Clínicas, Faculdade de Medicina da Universidade de São Paulo (HC-FMUSP) between January 1984 and December 2005. All the data on the patients were reviewed from their medical records.

To establish the diagnosis of PV according to the WHO criteria, the following investigations were carried out on all patients: physical examination, complete blood cell count, bone marrow trephine biopsy, arterial oxygen saturation, serum ferritin levels and hemoglobin electrophoresis. Additional tests, such as sleep studies, pulmonary function tests, peripheral blood or marrow karyotyping and serum erythropoietin analysis were performed as needed. All patients with borderline hemoglobin (Hb) levels according to the WHO criteria, and hematocrit above 52% for males and 48% for females, who had other signs of chronic myeloproliferative disorder (splenomegaly, leukocytosis or thrombocytosis), were evaluated by means of a radionuclide red cell mass test.^10^ This had the aims of confirming the diagnosis of PV and ruling out myelofibrosis and essential thrombocythemia.

### Clinical, laboratory and demographic characteristics

The following variables were evaluated at diagnosis: age, gender, hemoglobin concentration (Hb), platelet count, white blood cell count and history of cardiovascular complications, i.e. deep vein thrombosis (DVT), pulmonary embolism (PE), acute myocardial infarction (AMI), coronary insufficiency (CI), stroke, transient ischemic attack (TIA) or arterial thrombosis.

The presence of cardiovascular risk factors, i.e. high blood cholesterol levels, presence of diabetes mellitus (DM) or hypertension and smoking habits, and the presence of cytogenetic aberrations, was also evaluated.

The patients were stratified into two risk groups: low risk (age ≤ 60 years and absence of previous thrombosis) and high risk (age > 60 years or previous thrombosis).

### Definition of the main outcome events during follow-up

- Overall survival (OS) – defined as the time elapsed from the diagnosis of PV until death due to any cause or until loss from follow-up;
- Thrombosis-free survival (TFS) – defined as the time elapsed from the diagnosis of PV until the first thrombotic event (DVT, PE, arterial thrombosis, AMI, CI, stroke or TIA);
- Leukemia-free survival (LFS) – defined as the time elapsed from diagnosis of PV until diagnosis of sAML. The diagnosis of sAML was defined according to WHO criteria, by the presence of at least 20% blasts in bone marrow or peripheral blood;
- Myelofibrosis-free survival (MFS) – defined as the time elapsed from diagnosis of PV until secondary myelofibrosis. Secondary myelofibrosis was defined according to PVSG criteria, by the presence of marrow fibrosis detected by means of bone marrow biopsy and the presence of one of the following characteristics in patients with splenomegaly or four of these in the absence of splenomegaly: anisopoikilocytosis with teardrop erythrocytes, immature myeloid cells in peripheral blood, erythroblasts in peripheral blood, abnormal megakaryocyte clusters in bone marrow, or presence of myeloid metaplasia.

### Statistical analysis

Survival was analyzed by the Kaplan-Meier method, and comparisons were made using the log-rank test. All p values were two-tailed and the alpha error was defined as 5%. The statistical analyses were performed using the Statistical Package for the Social Sciences (SPSS) 10.0 for Windows (SPSS Inc, Chicago, United States).

## RESULTS

Seventy-two patients with PV were identified. Of these, six did not have adequate medical records and were excluded. The remaining 66 patients were evaluated.

The median age of the entire group of patients at diagnosis was 61 years old. Seven patients (10.6%) were less than 40 years old, 23 (34.8%) were between 40 and 60 years old and 36 (54.5%) were more than 60 years old. Thirty-six patients (54.5%) were female. The median Hb value at diagnosis for the whole group of patients was 18.8 mg/dl (range: 12.6 mg/dl to 21.7 mg/dl). For the male patients, Hb was 19.20 mg/dl (range: 13.8 mg/dl to 21.7 mg/dl) and for the female patients it was 17.4 mg/dl (range: 12.6 mg/dl to 21.6 mg/dl). Fifty-two patients (78.8%) had Hb levels above those defined by the WHO criteria. Among the other 14 patients (21.2%), three had low Hb levels due to concomitant iron deficiency that was corrected before a diagnosis was made and 11 had borderline Hb levels (median Hb value of 17.8 mg/dl for males and 16.0 mg/dl for females). All of these 11 patients could be classified as PV because they presented elevated red cell mass. Fifty-one patients (77.2%) had leukocytes counts of more than 12,000/mm^3^. Fifty-two patients (78.8%) had platelet counts of more than 400,000/mm^3^. Twenty-seven patients (40.9%) had a positive history of arterial thrombosis, while five (7.5%) had a positive history of venous thrombosis (Table 2).

**Table 1.** World Health Organization (WHO) diagnostic criteria for polycythemia vera (PV)

**Major**	
**A1**	Elevated red cell mass (> 25% above mean normal predicted value) or hemoglobin > 18.5 mg/dl in men or 16.5 mg/dl in women
**A2**	No cause of secondary erythrocytosis and absence of familial erythrocytosis
**A3**	Splenomegaly
**A4**	Clonal genetic abnormality other than Philadelphia chromosome or *Bcr/Abl* fusion gene in marrow cells
**A5**	Endogenous erythroid colony formation in vitro
**Minor**	
**B1**	Platelet counts > 400 × 10^9^/l
**B2**	Leukocytosis > 12,000/µl
**B3**	Bone marrow biopsy showing panmyelosis with prominent erythroid and megakaryocytic proliferation
**B4**	Low serum erythropoietin levels

**Note:** PV diagnosis is established by the following combinations: A1 + A2 + A3 or A4; and A1 + A2 + two of B.

**Table 2.** Patients’ characteristics

	n = 66 (%)
**Age**	
Median (range)	61 (26 – 86)
**Sex**	
Female	36 (54.5)
Male	30 (45.5)
**Hb at registration**	
Median (range)	18.8 mg/dl (12.6 – 21.7)
**WBC count**	
Median (range)	13,550/mm^3^ (6700 – 48780)
**Platelet count** *	
Median (range)	593,000/mm^3^(150,000 – 2,764,000)
**Previous arterial thrombosis**	
AMI	10 (15.1)
CI	3 (4.5)
Stroke	9 (13.6)
TIA	1 (1.5)
Peripheral	4 (6.0)
**Previous venous thrombosis**	
DVT	2 (3.0)
PE	0
Intra-abdominal thrombosis	2 (3.0)
Other	1 (1.5)
**Thrombotic risk factors**	
Arterial Hypertension‡	41 (66.1)
DM	8 (12.7)
Dyslipidemia§	16 (27.1)
Smoker||	22 (51.1)

Note:

* no data from four patients;

‡ no data from three patients;

§ no data from seven patients;

|| no data from 24 patients.

Hb = hemoglobin; WBC = white blood cell; AMI = acute myocardial infarction; CI = coronary insufficiency; TIA = transient ischemic attack; DVT = deep vein thrombosis; PE = pulmonary embolism; DM = diabetes mellitus.

The patients were stratified into two risk groups in relation to thrombosis. Twenty patients (30.3%) were classified as low risk and 46 (69.7%) as high risk.

Cytogenetic analysis was performed on 29 patients (43.9%). Of these, six (20.7%) had chromosomal abnormalities. The clonal chromosomal abnormalities found were: del6q, +9, add(18)(p11), t(1;11), -15 with -20 and del9.

### Treatment

After the diagnosis was made, all the patients were treated with phlebotomy to achieve a target hematocrit of 45% in males and 43% in females. To lower or do away with the need to keep on repeating the phlebotomy treatment, all of the high-risk patients and most of the low-risk patients were additionally treated with myelosuppressive agents to maintain the target hematocrit level (Table 3). Only one patient was treated with phlebotomy alone and three were treated with interferon. Forty-three patients (65.1%) also received low-dose aspirin for at least some part of the follow-up because of the presence of a previous arterial event or for primary prophylaxis of arterial occlusion in cases with thrombocytosis (Table 3).

**Table 3.** Treatment

Treatment	n = 66 (%)
Phlebotomy alone	1 (1.5)
**Chemotherapy +/- phlebotomy**	
Hydroxyurea	58 (88.0)
Interferon	3 (4.5)
Busulfan	3 (4.5)
Radioactive ^32^phosphorus	1 (1.5)
**Low-dose aspirin**	43 (65.1)

### Response to treatment

**Figure 1.**
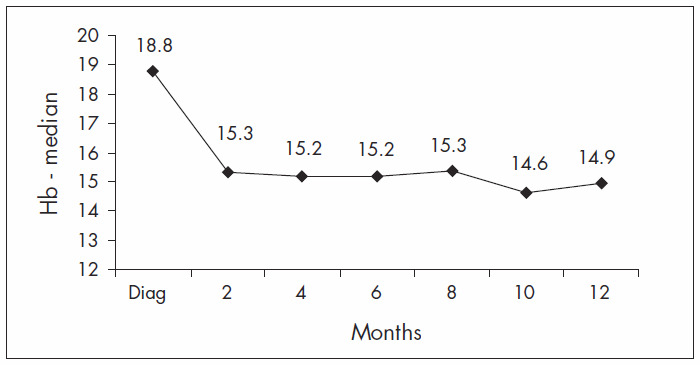
Median hemoglobin concentration (Hb; mg/dl), first-year bimonthly follow-up.

**Figure 2.**
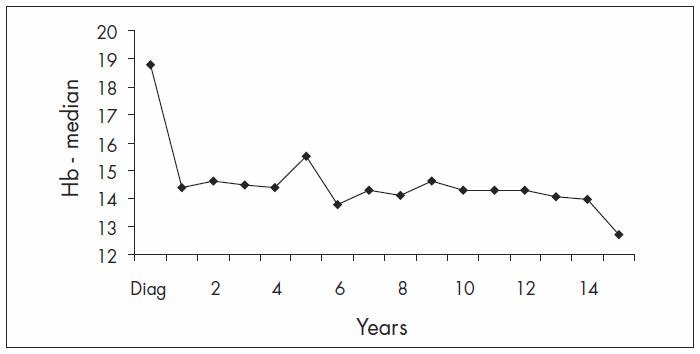
Median hemoglobin concentration (Hb; mg/dl), yearly follow-up.

Hb levels were observed during the follow-up. At diagnosis, the median Hb was 18.8 g/dl, with a progressive fall during the first year of treatment (Figure 1). After the first year, the median Hb levels were stable, ranging from 13.0 to 15.5 g/dl (Figure 2).

### Events during follow-up

The median follow-up for the entire cohort was 77.5 months (95% confidence interval, CI: 53.6 – 101.4 months). Thirty-nine patients (59.1%) did not have any event (thrombosis, hemorrhage, sMF, sAML or death). Twenty-two patients (33.3%) had thrombotic events: 15 (22.7%) had arterial thrombotic events and seven (10.6%) had venous thrombotic events. Some patients had more than one thrombotic event during their follow-ups. Only two (3%) progressed to sAML and five (7.5%) to secondary myelofibrosis (Table 4).

**Table 4.** Events during follow-up

Event	n = 66 (%)
**Arterial thrombosis**	
AMI	2 (3.0)
CI	3 (4.5)
Stroke	3 (4.5)
TIA	4 (6.0)
Peripheral	3 (4.5)
**Venous thrombosis**	
DVT	5 (7.5)
PE	1 (1.5)
Intra-abdominal thrombosis	1 (1.5)
**Bleeding**	
Yes	1 (1.5)
**Transformation**	
Acute leukemia	2 (3.0)
Myelofibrosis	5 (7.5)
**Death**	
Due to thrombosis	4 (6.0)
Other causes	5 (7.5)

**Note:** Some patients had more than one event during follow-up.

AMI = acute myocardial infarction; CI = coronary insufficiency; TIA = transient ischemic attack; DVT = deep vein thrombosis; PE = pulmonary embolism.

### Overall survival (OS)

**Figure 3.**
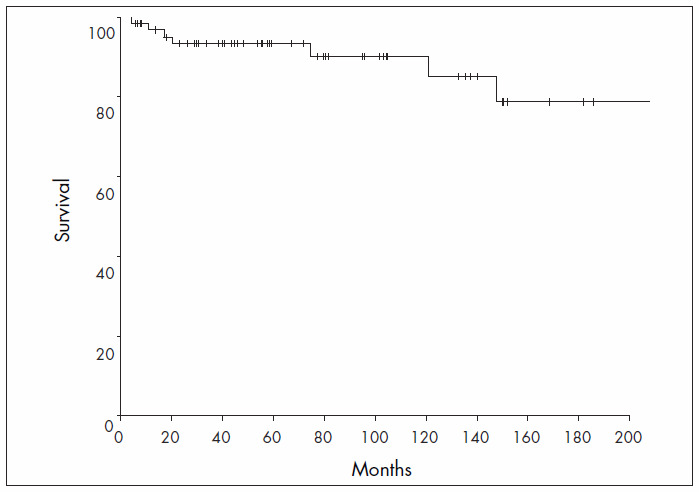
Overall survival.

The median OS was not reached, while the mean OS was 296.6 months (95% CI = 231.9 – 361.2 months), with a seven-year OS rate of 77.8% (Figure 3). The overall mortality rate was 1.9% per patient-year. The only factor associated with survival was age at diagnosis: patients older than 60 years of age had worse survival than did those younger than 60 (Table 5).

**Table 5.** Overall survival (OS), from univariate analysis

Prognostic variable	OS rate over seven years %	p
**Previous thrombosis**		
Yes	89.4	0.40
No	91.7	
**Age**		
Less than 60 years old	88.7	**0.01**
More than 60 years old	56.0	
**Other cardiovascular risk factors**		
Present	63.7	0.07
Absent	100.0	

### Thrombosis-free survival (TFS)

**Figure 4.**
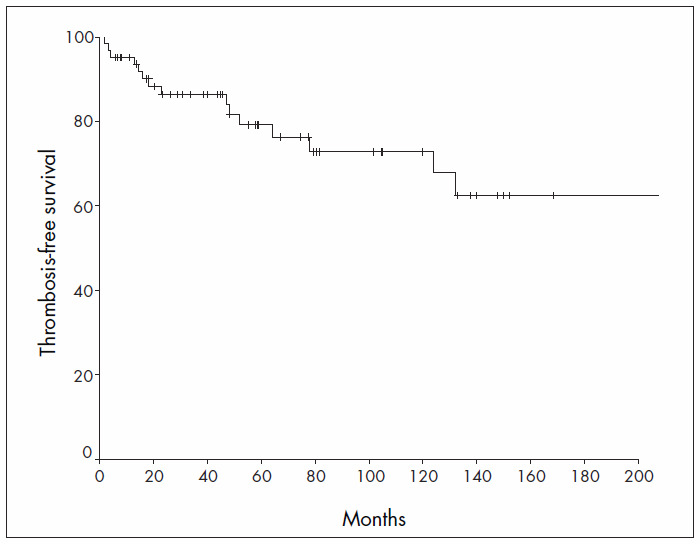
Thrombosis-free survival.

The median TFS was not reached, while the mean TFS was 245.2 months (95% CI = 183.6 – 306.9 months) and the seven-year TFS rate was 72.3% (Figure 4). The overall incidence of thrombosis was 5.5% per patient-year. Univariate analysis showed that none of the factors analyzed were associated with the presence of a thrombotic event (Table 6).

**Table 6.** Thrombosis-free survival (TFS), from univariate analysis

Prognostic variable	TFS rate over seven years %	p
**Previous thrombosis**		
Yes	66.9	0.26
No	63.9	
**Age**		
Less than 60 years	73.0	0.56
More than 60 years	73.3	
**Other cardiovascular risk factors**		
Present	56.6	0.88
Absent	65.2	

### Leukemia-free survival (LFS)

The median LFS was not reached (> 77 months), while the mean LFS was 372.2 months (346.7 – 397.8), the seven-year LFS rate was 94.7% and the crude sAML incidence was 0.42% per patient-year. None of the three patients that were treated with the alkylating agent busulfan or another patient treated with radioactive ^32^phosphorus progressed to acute leukemia during the follow-up.

### Myelofibrosis-free survival (MFS)

The median MFS was not reached (> 77 months), while the mean MFS was 293.1 months (216.4 – 369.8), the seven-year MFS rate was 75.2% and the pooled sMF incidence was 1.06% per patient-year.

## DISCUSSION

The incidence of PV in the United States and Europe ranges from 5 to 26 per million habitants. However, the incidence of PV in Brazil is unknown. The male-to-female (M:F) ratio of 1:1 observed in our center is similar to that found in trials carried out in other countries. In addition, the median age at diagnosis in our population (61 years) was similar to the median of 65.4 years found in the European study, which included more than 1600 patients.^11^

Among the other risk factors, there were no significant differences between the European and Brazilian patients. Marchioli et al.^11^ observed a previous arterial thrombotic event in 38.6% of their patients and a venous thrombotic event in 13.7%. In the present study, 40.9% and 7.5% of our patients had a previous history of arterial and venous thrombotic events, respectively.

The hemoglobin levels in our patients at diagnosis ranged from 12.6 mg/dl to 21.7 mg/dl. Some patients had Hb levels lower than those defined by the WHO criteria for diagnosing PV, but they presented elevated red cell mass that confirmed the absolute erythrocytosis and enabled the diagnosis of PV. This could be explained by the detection of iron deficiency in some patients, which was corrected later on, or elevated plasma volume, which can artificially decrease the hemoglobin concentration. We agree with the observations of Green et al.^12^ that the Hb levels included in the WHO criteria are not always precisely related to absolute erythrocytosis. We also believe that if only the WHO criteria are going to be used in a suspicious case with a borderline Hb level, a therapeutic trial with iron supplements or measurement of the red cell mass may be warranted, in order to make an accurate diagnosis. On the other hand, the use of trephine bone marrow biopsy, serum erythropoietin, cytogenetic and molecular analysis, which are all included in the WHO criteria, can help in diagnosing PV and differentiating it from other causes of secondary erythrocytosis with a high degree of certainty.

Approximately 20% of our patients who had conventional cytogenetic analysis presented genetic abnormalities, according to published criteria.^13^

Most of our patients were treated with phlebotomy together with hydroxyurea, while three were treated with busulfan and only one with radioactive ^32^phosphorus. This can be explained by the fact that most of them were diagnosed with PV relatively recently, i.e. since the 1980s. Since that time, the increased risk of sAML caused by the use of alkylating agents has become well known, as have the benefits of using hydroxyurea. The latter can safely reduce erythrocyte counts and thrombosis risks, in comparison with phlebotomy alone. This widespread use of hydroxyurea was perhaps the reason why we found extremely low incidence of sAML. Concerning the evolution to myelofibrosis, some studies from other countries have shown sMF rates of about 10 to 30% over 10 years of cohort follow-up.^14,15^ Since treatment with hydroxyurea has not been shown to reduce the sMF rate, its occurrence in only five of our patients is probably a reflection of our stringent definition of sMF, since the detection of SMF was based on non-standardized criteria in the trials performed before 2001.^14,15^

Treatment with hydroxyurea and phlebotomy gave rise to good hematological control in our population. The Hb levels were brought under control within the first three months of therapy, with Hb levels ranging from 13.0 to 15.5 g/dl. After the first year of follow-up, the Hb levels remained stable, ranging from 14.0 to 15.0 g/dl. In addition to hematological control, long survival time was seen in this particular cohort, although we were able to observe an association between age over 60 and higher risk of death, similar to previous descriptions by other authors^4,11,16^ and as expected in the general population. None of the other known risk factors analyzed were associated with worse overall survival or thrombosis-free survival, in contrast with previous reports.^4,8,15^ This may have been due to our small sample size.

Furthermore, when our patients were treated with hydroxyurea and phlebotomy, we observed a thrombosis incidence of 5.5% per patient-year, which was the same as observed by Marchioli et al.^11^ in a multicenter study with non-standardized therapy. Arterial thrombotic events were the main complication, followed by DVT. Our study showed that although our patients were properly treated and had Hb levels that were under control, thrombotic events continue to occur over the course of time. It can clearly be seen that no plateau is reached on the TFS curve (Figure 4). The explanation for this is that the incidence of thrombosis in general populations increases with age and the same can occur with PV patients, even when they are correctly treated.^8,11,15^

Even with a small patient sample, we were able to see that our population of PV patients had characteristics and outcomes that were similar to those of patients treated in centers in the United States and Europe.^4,11,16^ Although application of the WHO criteria allows PV diagnoses to be made only in the presence of elevated Hb levels associated with the existence of other characteristics compatible with chronic myeloproliferative disorders, some patients may require red cell mass measurement in order to correctly establish the diagnosis. Recently, a recurrent somatic activating mutation (V617F) in JAK2 kinase has been described in chronic myeloproliferative disorders and has been found to be present in most PV cases.^17^ In addition to the function of JAK2 V617F as a possible new target for signal transduction therapy, provision of widespread availability of its detection in our country could be a step forward for identifying PV patients.

## CONCLUSIONS

The demographics and characteristics of Brazilian PV patients were similar to those described in studies from other countries. Treatment of PV by means of phlebotomy and non-alkylating myelosuppressive drugs made it possible to bring the abnormal erythroid proliferation under control, with low risk of disease transformation into secondary leukemia. In spite of this good disease control, the occurrence of new thrombotic events, and particularly arterial events, was the most common complication observed.
